# Functional distinction of hyphal compartments

**DOI:** 10.1038/s41598-017-06422-6

**Published:** 2017-07-20

**Authors:** Martin Tegelaar, Han A. B. Wösten

**Affiliations:** Microbiology, Department of Biology, Utrecht University, Padualaan 8, 3584 CH Utrecht, The Netherlands

## Abstract

Hyphae of higher fungi grow at their tips and are compartmentalized by porous septa that enable inter-compartmental cytoplasmic streaming. Woronin bodies discontinue cytoplasmic streaming by plugging the septal pores. Here, it was assessed whether apical compartments of *Aspergillus niger* sustain their own growth or whether their growth depends on subapical compartments. Hyphae of wildtype and the Δ*hexA* strain, lacking Woronin bodies, had a similar morphology and growth rate. A total of 58% and 17% of the hyphae continued growing, respectively, after dissecting the 2^nd^ compartment. Extension rate of the apical compartments that continued growing was not affected, even when the carbon or nitrogen source was limiting. Thus, apical compartments are self-sustaining in growth. It was also shown that the first 8 subapical compartments of the wildtype, but not of the Δ*hexA* strain, function as a backup system for growth by forming new branches when their apical neighbouring compartment has been damaged. This backup system is pivotal in nature because of the life style of fungi to continuously explore their surrounding substrate that may prove hostile.

## Introduction

Mycelia of filamentous fungi consist of interconnected hyphae that grow at their apices and that branch subapically. Hyphae of fungi belonging to the Ascomycota and the Basidiomycota are compartmentalized by septa. These cross-walls have a central pore of 50–500 nm^1–3^ that allows inter-compartmental and inter-hyphal cytoplasmic mixing. However, it was recently shown that mixing in *Aspergillus* is constrained by Woronin bodies that plug the septal pores^4, 5^. These peroxisome-derived organelles plug about 50% of the most apical septa. Plugging incidence increases sub-apically resulting in 100% closure after the 9^th^ compartment. From these results it was concluded that hyphal compartments transform from an unicellular to a multicellular system^5^. Plugging of septal pores thus abolishes indiscriminate mixing of cytoplasm, but selective transport is possible via transporters in the septal plasma membrane^6^.

The multicellular nature of the sub-apical part of hyphae explains why zones of a colony are heterogeneous with respect to gene expression, growth, and secretion^7–18^. Even neighboring hyphae can be heterogeneous in cytoplasmic composition^4, 7, 8, 14, 15, 18–24^. This heterogeneity is abolished in a Δ*hexA* strain^4^. This strain lacks Woronin bodies because it does not produce the main protein component of these peroxisome-like organelles^25^ and, as a consequence, septal plugging is rarely observed^4, 26^.

The fact that the apical compartments of *Aspergillus* hyphae form an unicellular system would agree with the concept of the peripheral growth zone defining the number of compartments that are needed to maintain the maximum hyphal growth rate^27^. This zone was described to consist of 11, 14, 6, and 13 compartments in the case of *Aspergillus niger, Aspergillus wentii, Aspergillus nidulans*, and *Penicillium chrysogenum*, respectively. We here however show that apical compartments of *A. niger* are self-sustaining in growth thus disproving the concept of the peripheral growth zone. The subapical compartments do have a function by acting as a back-up system for growth when an apical compartment is damaged.

## Results

### Apical compartments maintain their growth after laser-dissection

Light microscopy showed that the hyphal growth rate of the *A. niger* wild-type and Δ*hexA* strains was similar with a mean extension rate of 99 ± 5 and 93 ± 8 µm h^−1^, respectively. Length (358 ± 62 and 350 ± 64) and diameter (5.8 ± 0.2 and 6.0 ± 0.3) of the apical compartments of wild type and Δ*hexA* were also similar. Hyphae were laser dissected within the 2^nd^, 3^rd^, 4^th^, and 11^th^ compartment. Mean hyphal growth rate of both strains was not reduced when the filaments had been dissected in the 11^th^ compartment. In contrast, it was reduced by 39, 22, and 23% after dissecting the 2^nd^, 3^rd^, and 4^th^ compartment of wild-type hyphae, respectively. These values were higher for Δ*hexA* with 82, 47, and 49%, respectively.

A total of 42, 20, 22, and 9% of wild-type hyphae stopped growing, respectively, when they had been dissected in the 2^nd^, 3^rd^, 4^th^, or 11^th^ compartment (Table 1). The apical septum had been open in all hyphae that had stopped growing after dissection of the 2^nd^ compartment. Conversely, wild-type hyphae that remained growing had either a closed (20%) or open (80%) apical septum at the moment of dissection. The percentage of Δ*hexA* hyphae halting growth after dissecting the 4^th^ or 11^th^ compartment was similar to that of wild-type. In contrast, the percentage was higher when the 2^nd^ or 3^rd^ compartment was dissected. These data show that Woronin bodies protect hyphae from stopping growth after a subapical compartment is damaged.

**Table 1:** Proportion of wild-type and Δ*hexA* hyphae that continue growing after laser dissection and their mean growth rate (±confidence interval) before and after laser dissection.

		Intact compartment(s)				
		1	2	3	10	Control
**wt hyphae that continue growing**	Proportion	58%	80%	78%	91%	100%
	Growth rate before dissection	97 ± 5	100 ± 3	77 ± 3	88 ± 5	87 ± 2
	Growth rate after dissection	104 ± 5	97 ± 3	74 ± 4	87 ± 4	86 ± 3
**Δ** ***hexA*** **hyphae that continue growing**	Proportion	17%	53%	59%	100%	100%
	Growth rate before dissection	111 ± 12	113 ± 6	105 ± 5	116 ± 6	92 ± 2
	Growth rate after dissection	103 ± 17	112 ± 15	88 ± 8	112 ± 8	91 ± 3

Growth rate of wild-type and Δ*hexA* hyphae that remained growing after cutting in the 2^nd^, 3^rd^, 4^th^, or 11^th^ compartment was not affected (Table 1). Similar results were obtained when the C- or N-source was excluded from the liquid medium (Data not shown; for growth conditions see Material and Methods). These data show that reduction of the mean growth rate of dissected hyphae is solely due to the fraction of hyphae that stop growing after this damaging event.

### Predictors for continued growth of dissected hyphae

Growth rate of wild-type and the Δ*hexA* hyphae was normally distributed (Fig. S1). The relatively slow and fast growing hyphae within these normal distributions did not show differences in the incidence of continued growth after dissection in the second compartment (Data not shown). Thus, no causality was shown between continued growth and growth rate of the hyphae. The initial volume of wild-type apical compartments that continued or stopped growing was not significantly different (Table 2) and therefore was not a predictor of continued hyphal growth. In contrast, residual cytoplasmic volume in the apical compartment did differ after dissecting the 2^nd^ compartment. Similar results were obtained with Δ*hexA*. However, the cytoplasmic volume that was lost was 5.6- and 2.6-fold higher in the case Δ*hexA* hyphae that had stopped or continued growing after laser dissection, respectively, when compared to their wild-type equivalents (Table 2). Binary logistic regression showed that the length of the apical compartment is also a predictor for continued growth after laser ablation (Supplemental Text 1).

**Table 2:** Mean length, width, volume, and growth rate (±confidence intervals) of apical compartments of wild-type and Δ*hexA* before and after laser dissection in the 2^nd^ compartment.

		Apical length (µm)	Hyphal width (µm)	Growth rate (µm/h) Before After cutting		Apical volume (pl) Before After cutting		Volume lost (pl)	Volume remaining (%)
**wt**	Compartments that stopped growing	293 ± 61	6.3 ± 0.4	92 ± 7	0 ± 0	6.4 ± 1.7	3.9 ± 1.8	2.4 ± 1.7	62 ± 21
	Compartments that continued growing	363 ± 67	5.7 ± 0.2	97 ± 5	112 ± 6	9.2 ± 1.3	8.1 ± 1.4	1.1 ± 0.6	86 ± 7
**Δ** ***hexA***	Compartments that stopped growing	287 ± 47	5.9 ± 0.3	108 ± 7	6 ± 4	8.4 ± 1.8	2.3 ± 1	6.2 ± 1.5	29 ± 12
	Compartments that continued growing	524 ± 151	6.2 ± 0.9	119 ± 14	127 ± 21	16 ± 8.4	10 ± 7.8	6.3 ± 0.9	50 ± 26

Hyphae were selected that had their apical septum at a distance <or> 400 µm from the apex. These hyphae were dissected at 400 µm from the tip. 11% and 43% of the wild-type and Δ*hexA* hyphae with a septum <400 µm from the apex (i.e. these hyphae were cut in the 2^nd^ comprtment) stopped growing. In contrast, all wild type and Δ*hexA* hyphae with a septum >400 µm from the apex (i.e. these hyphae were cut in the 1^st^ compartment) stopped growing. Cutting within the first compartment at 400 µm from the apex resulted in a loss of 49% and 68% of the cytoplasm for Δ*hexA* and wild-type hyphae, respectively, while 46% and 32% was lost when the dissection site at 400 µm from the tip was within the second compartment. These results show that the presence of a septum between the apex and the site of dissection, independent of the state of the septum, is the best predictor whether a hyphae continues its growth after ablation.

### Disruption of the Spitzenkörper is linked to the fate of hyphal fragments

**Figure 1:**
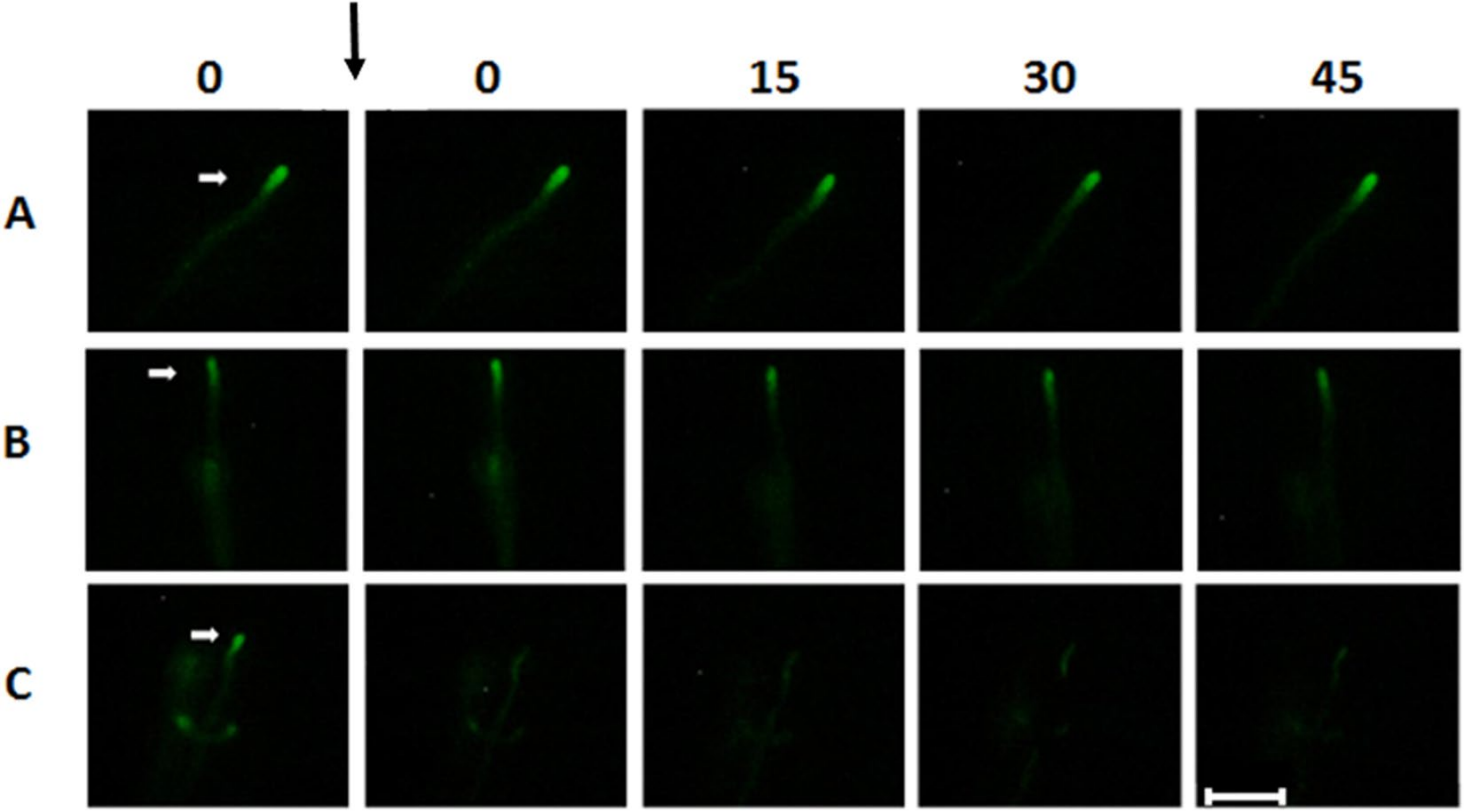
Fluorescently labelled Spitzenkörper before and after dissection in the 2nd compartment in a control hypha (**A**), a hypha continuing (**B**) and a hypha stopping growth (**C**) after dissection. Numbers indicate the time after laser dissection. White arrows denote the tip for which fluorescence was recorded. The black arrow indicates the moment of cutting. Bar represents 25 µm.

The Spitzenkörper of *A. niger* FG7 is fluorescently labelled due to a eGFP:: SncA fusion protein^28^. Surface area, length, and fluorescence intensity of the Spitzenkörper in the apical compartment was determined before and after dissecting the 2^nd^ compartment. Surface area, length, and fluorescence intensity of the Spitzenkörper of hyphae that stopped growing after dissection were smaller when compared to those of hyphae that remained growing (Fig. 1; Supplemental Table 1). These differences were not observed prior to dissection. These data show a relation between continued growth after dissection of the 2^nd^ compartment and integrity of the Spitzenkörper.

### Subapical compartments reinitiate growth after ablation of their apical neighbouring compartment

Branching incidence was assessed for compartments 2, 5, 8, and 10 of wildtype and Δ*hexA* hyphae that had either or not been cut in the apical neighbouring compartment. Branching in subapical compartments was hardly (<2% for the 2^nd^ compartment), if at all (for the 5^th^, 8^th^, and 10^th^ compartment), observed when Δ*hexA* hyphae had been dissected in their neigboring apical compartment. In contrast, dissection of compartment 1 of wild-type hyphae increased branching frequency of compartment 2 from 30% to 93% within a 4 h period (p ≤ 0,05). Similarly, dissection of compartment 4 or 7 increased branching incidence from 29% to 71% and from 2% to 31%, respectively. Branching incidence of compartment 10 did not increase significantly (0% versus 4.5% without and with cutting) when hyphae were dissected in compartment 9. Branching incidence in compartments 2 and 5 was statistically higher than branching incidence in compartments 7 and 9 for both cut and uncut wild-type hyphae. Together, these data show that sub-apical compartments act as a back-up for growth when apical compartments are damaged. This back-up is not functional in the absence of Woronin bodies. Moreover, the back-up capacity of hyphal compartments diminishes when compartments are further away from the hyphal tip.

## Discussion

Septate fungal hyphae like those of *A. niger* have been reported to require a minimal length of 1200 µm^29^ or 11 intact compartments^27^ to allow the maximum growth rate. We here however showed that apical compartments (mean length 354 µm) sustain their own maximum growth rate. This discrepancy is probably explained by the methodology adopted. Lhoas^29^ and Trinci^27^ damaged multiple hyphae with a metal blade and growth rate was expressed as the increase in colony diameter. In contrast, we dissected single hyphae with a laser and mean growth rate was determined taking into account the heterogeneity between the leading hyphae at the edge of the colony. In the case of the wild-type 42% of the hyphae stopped growing after dissecting the second compartment. These hyphae reduced the average growth rate of the whole population of hyphae. At the same time, average growth rate was not affected of those hyphae that remained growing after dissecting the second compartment. This shows that apical compartments do not depend on subapical compartments to sustain their growth.

The autonomy of apical compartments raised the question what the function is of subapical compartments. Subapical compartments may have a feeding function under nutrient deprivation, which was suggested from the finding that glucose is transported from the center to the periphery of the *A. niger* colony^6^. Yet, in our experimental set up apical compartments were still autonomous when N- and C-sources were limited. We did show that subapical compartments function as a back-up system. All hyphae stopped growing when apical compartments were dissected. This was accompanied by an increased branching incidence of the second compartment. These branches formed new exploring hyphae. Similarly, branching incidence was increased in compartment 5 and 8 after dissection of compartments 4 and 7, respectively. Branching incidence of compartment 10 was low, if observed at all, after dissecting compartment 9. This implies that this compartment and other more subapical compartments have lost their capacity to reinitiate growth. The ability of subapical compartments 2–8 to restore hyphal growth after their apical neighboring compartment has been damaged is pivotal considering the fungal life style. Mycelia continuously extend by colonizing their surrounding substrate. Apical compartments of exploring hyphae are the first to be confronted with microbes or other organisms within the non-explored substrate that can damage or feed on hyphae. Subapical branching combined with extension at an angle of the axis of the damaged hyphae provides a way to escape these organisms.

Woronin bodies can close septal pores, thus preventing cytoplasmic bleeding after hyphal damage^25, 30–32^. In addition, these organelles maintain inter-hyphal and inter-compartmental heterogeneity of cytoplasmic composition^4^. We here showed that Woronin bodies are not involved in the growth rate of hyphae before or after ablation but we found two additional functions of these organelles. First, branching in the sub-apical compartment was not observed in Δ*hexA* hyphae after ablation of the apical neighboring compartment. This shows that Woronin bodies are essential for the sub-apical back-up system of growth. Second, Woronin modies make hyphae more resilient to loss of cytoplasm. Half of wild-type hyphae were calculated to remain growing when 61% of the cytoplasmic volume in the apical compartment is retained, while 76% was needed in the case of Δ*hexA* hyphae. The resilience of the wild-type to cope with loss of cytoplasm is likely due to its ability to plug the septal pore, thus increasing the chance of the apical compartment to regain turgor. Experimental evidence indicates that not only loss of cytoplasm but also (partial) disintegration of the Spitzenkörper may result in halting of growth. Although in many cases occurrence of these processes will correlate, this is not necessarily the case. For instance, a strong but short outflow of cytoplasm may cause disintegration of the Spitzenkörper but would retain a sufficient amount of cytosol.

The observation that hyphae of *A. niger* always stop growing when the apical compartment is damaged implies that positioning of a septum close to the tip reduces the chance of abolished growth simply because the length available for a fatal damage would be less. In fact, fungi may increase septal incidence (i.e. reduce compartment length) to cope with stress. Indeed, length of apical compartments of *A. niger* is reduced at temperatures above 30 °C, coinciding with an increased branching incidence^33^.

Together, we have for the first time provided evidence that apical hyphal compartments of *A. niger* are autonomous with respect to growth. The subapical compartments are however metabolically active consuming a major part of resources. This drain of resources could be prevented by controlled autolysis of subapical compartments. This, however, would also result in the absence of the sub-apical back-up system of hyphal growth that is used when apical compartments become damaged.

## Materials and Methods

### Strains and growth conditions

strains N402^34^, Δ*hexA* ^26^, and FG7^28^ of *A. niger* were grown at 30 °C in water-saturated air at 700 lux white light (Osram Lumilux L36w/840, Osram, Munich, Germany). Spores were harvested in 10 ml 0.9% NaCl (w/v), 0.05% (v/v) Tween-20 from 7-day-old cultures grown in 9 cm Petri dishes on complete medium (CM) consisting of minimal medium (0.6% NaNO_3_, 0.15% KH_2_PO_4_, 0.05% KCl, 0.05% MgSO_4_ 7H_2_O, 0.2 ml^−1^ Vishniac solution^35^) with 0.2% tryptone, 0.1% casamino acids, 0.1% yeast extract, 0.05% yeast ribonucleic acids, 1.5% agarose, and 25 mM maltose. These spores were used to inoculate glass bottom dishes (MatTek Corporation, Ashland, MA, USA, P35G-1.5-20-C). To this end, the dishes and medium were pre-warmed to 50 °C and 60 °C, respectively. 0.5 µL spore solution (50,000 spores) was placed on a glass coverslip (18 mm in diameter and 0.16–0.19 mm thick) and left to dry. Pre-warmed CDMMA (30 µL) consisting of CD + Met medium (0.3% NaNO_3_, 0.2% KCl, 0.1% KH_2_PO_4_, 0.05% MgSO_4_.7H_2_O, 0.002% FeSO_4_.7H_2_O, 0.0015% methionine, pH 5.5^36^) with 1% agarose and 25 mM maltose was added on top of the glass bottom dish and immediately covered with the coverslip with the spores facing the medium. After the 118 µm thick layer of medium had solidified, 2 ml liquid CDMM was pipetted on top of the coverslip. CDMMA with 0.2% maltose and CDMM medium without a carbon source or CDMMA and CDMM without methionine were used to expose hyphae to C- or N-limiting conditions.

### Microscopy

Hyphae were dissected using a PALM Microbeam system linked to an Axiovert 200 inverted microscope (Carl Zeiss AG, Oberkochen, Germany) and a 3CCD color camera (HV-D30,Hitachi Kokusai Electric Inc., Tokyo, Japan). The glass bottom dish cultures were incubated at the microscope stage for 90 min at 25 °C before dissection. Hyphal growth rate within the peripheral zone of the colony was recorded every 5 min during a 45 min period, after which half of the hyphae were dissected in the 2^nd^, 3^rd^, 4^th^, or 11^th^ compartment using the laser pressure catapulting function of the PALM MicroBeam system (laser power 55%, focus 59%). At the moment of dissection it was assessed whether septal pores were open or closed. Septa were classified as open when cytosol was leaking through the septal pore upon dissection^4^. Hyphal growth was recorded every 5 min during a 45 min period immediately after dissection, non-dissected hyphae serving as control. Width and length of the compartment pre- and post-dissection were also recorded, as well as translocation (µm) of vacuoles after cutting. From these parameters the post-dissection volume of the apical compartment was calculated using *V*_*e*_ = *V*_*0*_ − *V*_*1*_, where *V*_*0*_ = 1/2 π (2/3 ${{\rm{d}}}_{0}^{3}$ + ${{\rm{d}}}_{0}^{2}$ [l − 1/2 d_0_]) and *V*_*1*_ = 1/2 π (2/3 ${{\rm{d}}}_{1}^{3}$ + ${{\rm{d}}}_{1}^{2}$ [l − 1/2 d_1_]) in the case volume was calculated based on reduction of hyphal diameter and where *V*_*0*_ = 1/2 π (2/3 ${{\rm{d}}}_{0}^{3}$ + ${{\rm{d}}}_{0}^{2}$ [l − 1/2 d_0_]) and *V*_*1*_ = 1/2d_0_^2^πt_l_ when volume was calculated based on translocation of vacuoles (Fig. S2).

Fluorescence was recorded using a HV-D30 camera with settings for white balance 5600 K; gain +8 db; contour correction high; shutter speed 1/50; digital gain +6 db; gamma on; contrast off; knee automatic. Total corrected cellular fluorescence was calculated with ImageJ. The surface of the Spitzenkörper was estimated by using the ImageJ threshold function using a brightness of 27.

### Statistics

Experiments were performed using biological duplicates (assessment of growth rate) or triplicates (plugging state,loss of volume, and renewed subapical growth). A one-way ANOVA with a Sidak post-hoc test or Kruskall-Wallis test with subsequent Wilcoxson rank sum tests was carried out to determine whether growth rate differed between hyphae with a different number of intact compartments. A χ^2^ test was performed with post-hoc z-tests and a Bonferroni correction for multiple comparisons to assess differences in plugging incidence between septa. The effect of plugging on hyphal growth was evaluated using one-way ANOVA and a Sidak post-hoc test, differences in hyphal growth before and after cutting were determined using paired sample t-tests. Differences in volume loss were statistically analyzed using two-way ANOVA, followed by correlation and regression analyses. Relative values pertaining to volume of apical compartments before and after cutting were analyzed using a paired sample t–test with and without logit transformations. Analysis of bimodality was performed as described^20^. Renewed growth from compartments located subapically from compartments damaged by laser dissection was analyzed by a χ^2^ test with post-hoc z-tests and a Bonferroni correction for multiple comparisons.

## Electronic supplementary material

Supplementary Information
