# Supplementary material for: Functional distinction of hyphal compartments
Source: Sci Rep. 2017 Jul 20;7:6039. doi: 10.1038/s41598-017-06422-6 (PMC5519613; doi:10.1038/s41598-017-06422-6)
Supplement: Supplementary file 1 — Supplementary Information [file 41598_2017_6422_MOESM1_ESM.pdf]

Supplemental Information

FUNCTIONAL DISTINCTION OF HYPHAL COMPARTMENTS

Martin Tegelaar and Han A. B. Wösten*.

Supplemental Text 1

Binary logistic regression predicted that 50 % of the Δ*hexA* hyphae would continue growing when 76 % of the volume remained in the apical compartment after dissecting the 2nd compartment. A remaining cytoplasmic volume ≥ 11.2 pl and ≤ 4.7 pl predicted that all Δ*hexA* hyphae would continue or stop growing, respectively. In contrast, a remaining volume > 2.7 pl predicted that 100 % of the wild-type hyphae would continue growing. Length of wild-type and Δ*hexA* apical compartments was also a predictor of continued growth with an accuracy of 88 % as determined by binary logistic regression. Both data sets predicted that for every 10 µm increase in length of the apical compartment the likelihood of stopping after dissecting the second compartment decreased by 7.5 %. All hyphae were predicted to continue growing with a compartment length of 77 µm for the wild-type and 629 µm for the Δ*hexA* strain.

Supplemental Table 1. Length, surface area, and fluorescent intensity (± confidence intervals) of the Spitzenkörper before and after laser dissection in the second compartment.

	Before cutting			After cutting		
	**Length (µm)**	**Surface ($µm^2$)**	**Fluorescence Intensity (au)**	**Length (µm)**	**Surface (µm)**	**Fluorescence Intensity (au)**
**Hyphae continuing growth**	9.2 ± 1.3	42 ± 7	1205 ± 123	7.2 ± 0.9	32 ± 4	1039 ± 70
**Hyphae Stopping growth**	6.2 ± 3,8	37 ± 22	1145 ± 297	3.8 ± 2,2	22 ± 14	766 ± 212

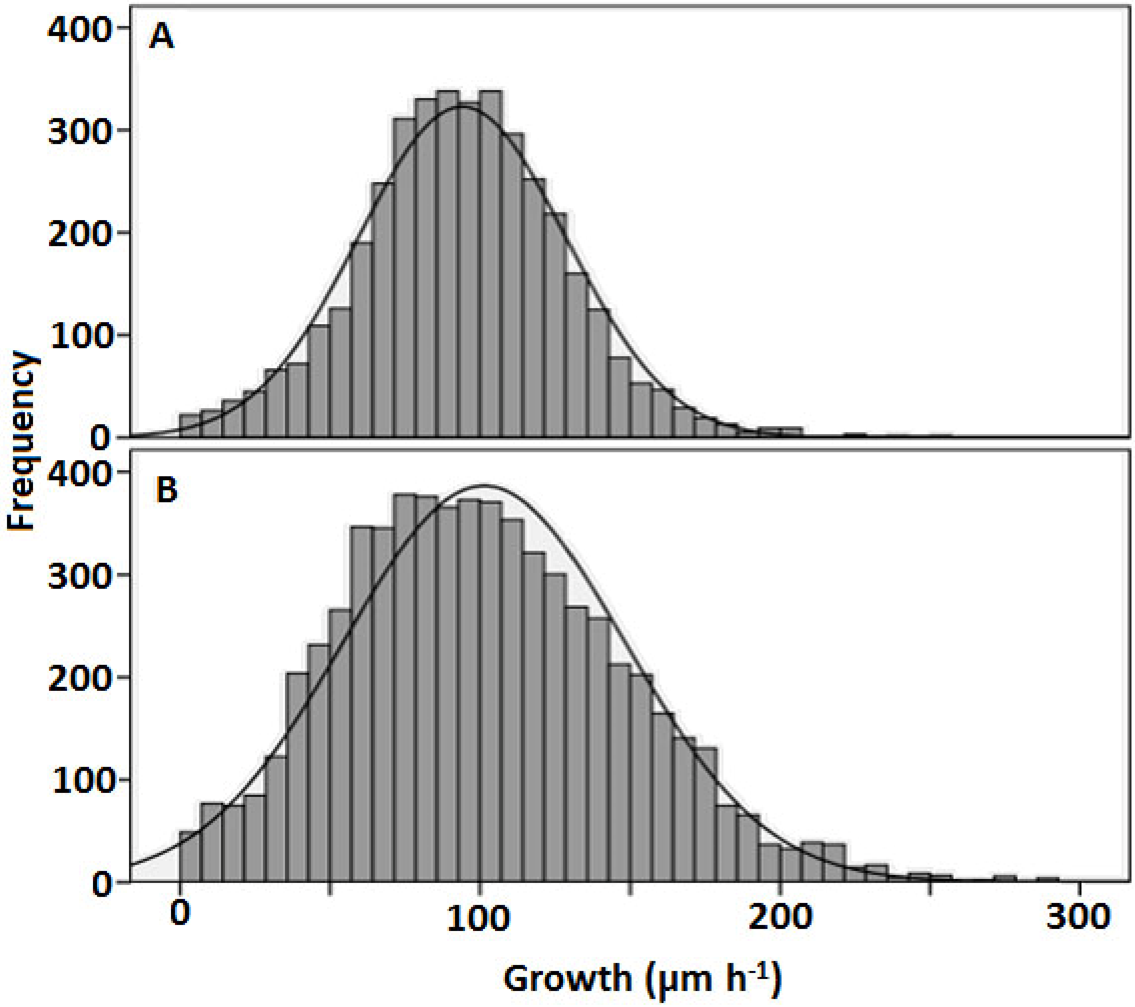

Supplemental Figure 1: Distribution of growth rate of wild-type and Δ*hexA* hyphae of *A. niger*.

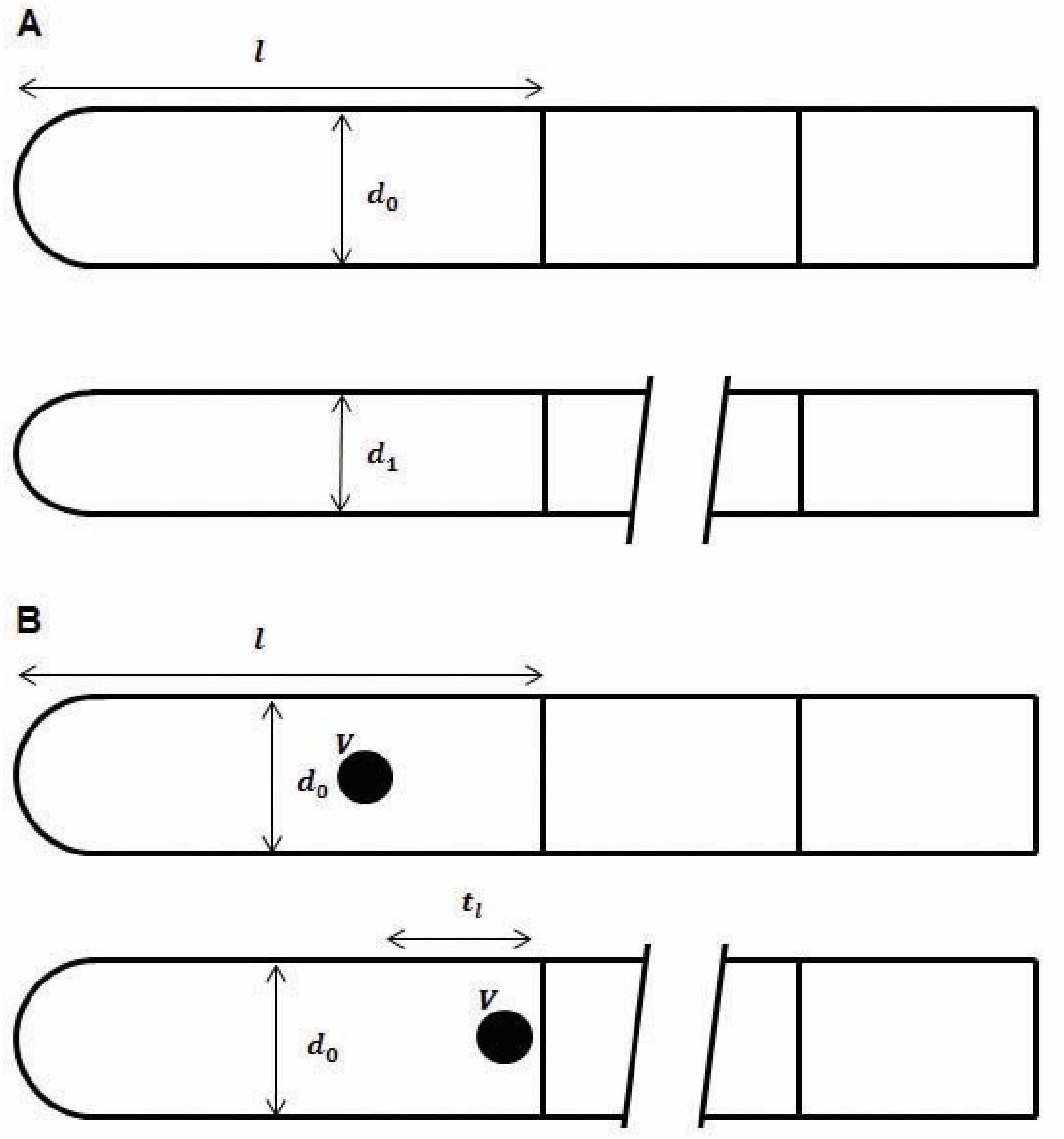

Supplemental Figure 2. Parameters used to calculate loss of cytosolic volume after dissection of a neighboring compartment. d_0 and d_1 denote the diameter of the compartment before and after dissection, respectively. l is the total length of the compartment, while l_t represents the distance vacuoles traveled after dissection.
